# A community-driven hypertension treatment group in rural Honduras

**DOI:** 10.3402/gha.v8.28041

**Published:** 2015-09-10

**Authors:** Sheridan Reiger, Jeffrey R. Harris, Kwun Chuen Gary Chan, Hector Lopez Oqueli, Marlana Kohn

**Affiliations:** 1 Department of Medicine, University of Washington School of Medicine, Seattle, WA, USA; 2 Department of Epidemiology, University of Washington School of Public Health, Seattle, WA, USA; 3 Salud Juntos, Seattle, WA, USA; 4 Health Promotion Research Center, Department of Health Services, School of Public Health, University of Washington, Seattle, WA, USA; 5 Department of Biostatistics, School of Public Health, University of Washington, Seattle, WA, USA; 6 Departmento de Yoro, Morazán, Hector Lopez Oqueli, Secretaría de Salud, Yoro, Honduras

**Keywords:** global health, community health workers, program evaluations, generic anti-hypertensives, group-based treatment

## Abstract

**Background:**

We formed a self-funded hypertension treatment group in a resource-poor community in rural Honduras. After training community health workers and creating protocols for standardized treatment, we used group membership fees to maintain the group, purchase generic medications in bulk on the local market, and hire a physician to manage treatment. We then assessed whether participation in the group improved treatment, medication adherence, and hypertension control.

**Design:**

This is a program evaluation using quasi-experimental design and no control group. Using data from the 86 members of the hypertension treatment group, we analyzed baseline and follow-up surveys of members, along with 30 months of clinical records of treatment, medication adherence, and blood pressure readings.

**Results:**

Our initial hypertension needs assessment revealed that at baseline, community hypertensives relied on the local Ministry of Health clinic as their source of anti-hypertensive medications and reported that irregular supply interfered with medication adherence. At baseline, hypertension group members were mainly female, overweight or obese, physically active, non-smoking, and non-drinking. After 30 months of managing the treatment group, we found a significant increase in medication adherence, from 54.8 to 76.2% (*p*<0.01), and hypertension control (<140/90 mmHg), from 31.4 to 54.7% (*p*<0.01). We also found a mean monthly decrease of 0.39 mmHg in systolic blood pressure (*p*<0.01). At the end of the 30-month observation period, the local Ministry of Health system had increased provision of low-cost anti-hypertensive medications and adopted the hypertension treatment group's treatment protocols.

**Conclusions:**

Formation of a self-funded, community-based hypertension treatment group in a rural, resource-poor community is feasible, and group participation may improve treatment, medication adherence, and hypertension control and can serve as a political driver for improving hypertension treatment services provided by the public system.

Long considered a problem of the ‘developed world’, hypertension is now the second leading risk factor for death and disability globally and in Honduras (1, 2). Treatment of hypertension in Latin American countries like Honduras is often complicated by inconsistent access to care and medications (3).

Initiatives to improve treatment of hypertension and other chronic diseases in low-resource settings have often included three evidence-based components: the participation of community health workers (CHWs), algorithm-driven use of generic medications, and group visits. CHWs, defined as ‘community health aides selected, trained, and working in the communities from which they come’, have participated in programs to improve self-management and outcomes for individuals with chronic conditions including hypertension and diabetes (4–8). Algorithm-driven use of first-line generic medications to treat hypertension has been found to be as effective as use of their brand name counterparts (9). Group visits are defined as medical appointments offered to a group of patients with the same medical condition instead of more traditional one-on-one visits (10). This format of care delivery, with its group dynamics, provides theoretical upsides in cost-effectiveness, time efficiency, peer-to-peer teaching, and peer-motivated behavior change (11). Group-visit interventions have shown improvements in glycemic and hypertension control (10, 12, 13).

The hypertension treatment program evaluated here belongs to the rural Honduran community of Punta Ocote, a rural town in central Honduras. It has 850 residents and a surrounding catchment area of 3,500 people served by its Ministry of Health (Ministry) clinic. Punta Ocote is a 30-minute bus ride away from the nearest private pharmacy. In 2010, the Ministry, the non-profit organization Salud Juntos, local hypertension patients, and health leaders agreed to form a group program to improve treatment of hypertension in Punta Ocote. The group's first meeting and treatment session took place in March 2010.

We conducted the program evaluation described here to assess the effect of the group program on the quality of hypertension care. To do so, we characterized baseline hypertension treatment and assessed trends in treatment provided, adherence, and blood pressure (BP).

## Methods

### Study population

Treatment group members initially consisted of individuals who had previously been treated for hypertension at the Ministry clinic. Members who joined the group after March 2010 were restricted to those individuals who were from the clinic's catchment area, had been screened by CHWs, and were diagnosed with hypertension by the group's physician.

### Evaluation design and ethics approval

Working with previously collected data from the hypertension treatment group, we utilized a quasi-experimental design and no control group to complete this program evaluation. The University of Washington Human Subjects Division granted the evaluation Research Exempt Status.

### Medication protocols and provision

Honduran and University of Washington physicians consulted with Salud Juntos to form simple, evidence-based protocols using medications from the Ministry formulary and following PAHO (14), ALLHAT (15), and JNC7 (16) guidelines. These protocols recommend a first-line, low-dose diuretic (hydrochlorothiazide), followed by stepwise addition of second-line generic anti-hypertensive medications including an ACE inhibitor (enalapril), beta-blocker (atenolol), and calcium-channel blocker (nifedipine). Low-dose aspirin use was also integrated into the protocols to lower the risk of ischemic heart disease (17).

The hypertension treatment group developed a disease-specific micro-insurance scheme to pay for medications and group expenses. Each group member paid a monthly membership fee of 50 Lempiras (~$2.60) to the group. This money was collected and managed by elected group leaders at monthly meetings of the group. The pooled funds were used to purchase bulk medications from an in-country wholesaler, contract with one visiting physician to tend to the group 6 hours per month, and cover administrative costs. The group physician was specifically contracted to manage hypertension care for group members during these monthly meetings. The membership fee was considerably lower than the costs associated with traveling to the closest private pharmacies and purchasing medications.

### Role of CHWs

Two CHWs from Punta Ocote played key roles in group coordination, member outreach, and data collection. The CHWs assisted the elected leaders of the group with convening meetings, medication ordering, and community promotion of the group. During monthly-meeting days, the CHWs gave hypertension education talks, led group discussions, helped dispense medications, took BP readings, and asked a set of triage questions of each member to help prioritize which members should be seen by the group physician. CHWs were trained in these capacities by Salud Juntos volunteers and local Ministry nurses and were paid a salary in accordance with Honduran Labor Law by Salud Juntos. CHWs did not hold any certifications, as none exist in Honduras.

### Community hypertension needs assessment

Prior to beginning the group program, we assessed hypertension-related needs in Punta Ocote by administering a modified World Health Organization STEPS Survey (18) to community members who had previously been diagnosed with hypertension and were followed by the Ministry clinic.

### Assessment of outcomes

Our assessment of changes in BP, medication adherence, and meeting attendance relies on a chart review of group-member forms completed monthly. All BPs were measured in accordance with JNC7 and PAHO protocols – allowing for 5 min of seated rest before BP was measured with arm at heart level, feet on the ground, preferring right arm unless contraindicated. Medication adherence was assessed with the question: ‘Have you taken your hypertension medications in the last 24 hours?’

We restricted our outcomes analysis to community members who had hypertension and were enrolled in the treatment group for 3 or more months within the 30-month analysis period, regardless of enrollment status at the end of the collection period. Comparison of initial and final measurements used values recorded at the first month of a member's treatment and the most recently recorded values for that member. Initial versus final comparisons for systolic blood pressure (SBP) and diastolic blood pressure (DBP) were tested with paired t-tests. We used McNemar's test of paired data to compare initial and final proportions of members who were adherent with treatment, on multidrug therapy, taking hydrochlorothiazide, and taking low-dose aspirin and had controlled hypertension (<140/90 mmHg).

### Attendance and dropout

Meeting attendance was recorded by group CHWs and used to generate enrollment duration (months between initial and final month) and total monthly-meeting visits. Member who did not attend during the most recent 3 months of the data-collection period (June, July, and August 2012) were classified as drop-outs.

### Regression modeling

We constructed random-effects models to identify trends in BP and BP control by months of participation. We fitted a linear random-effects model regression with combined random-intercept and random-slope for both SBP and DBP using the primary predictor of months of group participation. A logistic regression random-effects model for BP control (<140/90 mmHg) used the primary predictor of months of group enrollment. For both models, we tested the influence of other covariates and, finding none, included only age and sex.

### Community engagement

Our objectives for community engagement were evaluated by the initiation, development, set-up, and sustainability of the hypertension treatment group through reporting of activities and experiences by both Salud Juntos CHWs and employees of the local Ministry clinic.

## Outcome

### Community hypertension needs assessment

Prior to group establishment, most hypertensive patients responding to the needs-assessment survey (*n*=75) were middle-aged, female (77%), overweight or obese, non-smokers, non-drinkers, and physically active and had low fruit and vegetable intake (Table 1). Most reported accessing anti-hypertensive medications locally through the Ministry system (72%). Most reported that they had poor access to these medications over the past year (79%) and that this poor access resulted in limited treatment adherence.

**Table 1:** Demographics, behaviors, and hypertension history of respondents to community hypertension needs assessment (*n*=75)

	Mean	SD
Age in years at baseline	58.81	13.04
Years of formal education	2.72	2.71
	Frequency	Percent
Female sex	58	77.3
BMI category		
Healthy weight	15	23.8
Overweight	27	42.9
Obese	21	33.3
Smoking		
Non-smoker	56	77.8
Former smoker	14	19.4
Current smoker	2	2.8
Alcohol use^a^		
Non-drinker	55	87.3
Non-binge drinker	6	9.5
Binge drinker	2	3.2
Fruit portions per day		
0–2	71	94.7
3–4	4	5.3
Vegetable portions per day		
0–2	75	100
Physical activity^b^		
Sedentary	10	13.7
Active	63	86.3
Diabetes diagnosis history		
Not tested	26	35.1
Tested, no diabetes	31	41.9
Tested, has diabetes	17	23

SD, standard deviation; BMI, body mass index. Any totals for responses which do not add up to 75 represent missing data.

a Drinker defined as self-reported use of alcohol within previous year. Binge drinker defined as self-reported consumption of ≥5 drinks in a sitting for males and ≥4 for females within last 30 days per WHO STEPS definitions.

b Sedentary defined as no self-reported participation in exercise activity in an average week.

### Assessment of outcomes

The 86 community members who enrolled in the group program were similar to the respondents to the needs-assessment survey. The program members were slightly older (mean age 59.6 years), but like the baseline group were mostly female (80%), and overweight or obese (81%). By August 2012, 47 of the 86 members (55%) had dropped out, with men (29%) less likely than women (61%) to drop out. Mean participation length was 16.85 months (range, 3–30 months).

Comparing data from the first and final months of participation, we found significant improvement in hypertension control and treatment adherence within the hypertension group (Table 2). Among group members, mean SBP decreased significantly during participation, while DBP did not. The linear random-effects model estimated a mean SBP decrease of 0.39 mmHg (−0.56 to −0.22; *p*<0.01) and a mean DBP decrease of 0.07 mmHg (−0.19 to 0.04; *p*=0.21) per month of participation. The logistic random-effects model for BP control estimated an increase of 4.2% (2.2%–6.1%; *p*<0.01) in the odds of having a controlled BP, per month of enrollment.

**Table 2:** Initial and final measurements for hypertension group members (*n*=86)

	Initial mean (95% CI)	Final mean (95% CI)	
Systolic BP, mmHg	151 (145,155)	140 (136,145)	
Diastolic BP, mmHg	85 (83, 88)	83 (80.8, 85)	
Control and adherence	Initial proportion (%)	Final proportion (%)	*p*
BP<140/90	31.4	54.7	<0.01
BP<160/100	68.6	86.0	<0.01
Medication adherence	54.8	76.2	<0.01
Medications	Initial proportion (%)	Final proportion (%)	*p*
Taking hydrochlorothiazide	36.0	53.5	<0.01
Taking>1 medication	33.7	48.8	<0.01
Taking aspirin	60.5	75.6	<0.01

BP, blood pressure; CI, confidence interval. Description: Table of comparisons of initial and final blood pressure and treatment statistics gathered from monthly reporting of a community hypertension treatment group in Punta Ocote, Honduras. Initial and final comparisons showing *p* values were tested using McNemar's test for significance. Hydrochlorothiazide, combination pharmacologic treatment for hypertension, and low-dose aspirin were all assessed to identify possible change in conformity to treatment protocols.

### Community engagement

According to local nurses and CHWs, the presence of affordable and effective hypertension care through the group program led local Ministry officials to ‘compete’ with these services. Specifically, the Ministry clinic adopted the program's treatment protocols and began to more consistently source anti-hypertensive medications. As a result, much of the dropout which we identified in this evaluation is likely to be attributable to members’ reversion to Ministry care.

## Interpretation

The evaluation showed that the hypertension treatment group program increased medication adherence, lowered BP, and increased hypertension control for its members. The longevity of the program, along with many positive reviews by its members, further supports its value. Additionally, the effect on the provision of care by the Ministry system was both surprising and encouraging. It indicates a capacity for improvement within a government health system when an outside impetus is applied.

There are several lessons to be learned from the development and implementation of this program. First, the effects on the provision of Ministry care revealed that in future implementations of similar programs, the development of stronger communication and frank discussion of themes, including patient- and resource-sharing, should be built into the planning phase of implementation. Second, though no life-threatening negative effects from medications were detected, it would be appropriate to identify ways to embed electrolyte and creatinine testing and orthostatic BP assessment into treatment protocols. Third, the use of CHW home visits should have been anticipated and built into the program.

Our evaluation has several limitations. First, data were gathered from a program implementation, and not from a research study. Therefore, there are no comparison groups and only baseline to compare against. Second, our sample size is small, and this diminishes our ability to find an effect of program participation. Third, the member dropout rate was high, and we were not able to obtain follow-up data on those who dropped out.

Our evaluation supports the continued testing and evaluation of the use of generic anti-hypertensive medications and CHWs for chronic disease treatment in low-resource settings like Honduras. At this time, there are a very limited number of outcomes-based investigations for interventions to improve hypertension care in low-resource settings. Therefore, we are unable to make comparisons to existing literature. The use of a disease-specific micro-insurance scheme to pay for medications and other group treatment-related expenses is unique in the literature, and we feel that this self-pay mechanism for medication provision warrants further testing in other settings. The use of group visits in low-resource settings is also not well documented, and its use in this effective program indicates its potential in contexts similar to rural Honduras.

In summary, our evaluation showed that the development of a sustainable mechanism for provision of medication and consistent care using CHWs can increase treatment adherence, improve hypertension control, and have a positive community impact.
